# A measure to evaluate deformable registration fields in clinical settings

**DOI:** 10.1120/jacmp.v13i5.3829

**Published:** 2012-09-06

**Authors:** Eduard Schreibmann, Paul Pantalone, Anthony Waller, Tim Fox

**Affiliations:** ^1^ Department of Radiation Oncology Emory University School of Medicine Atlanta Georgia USA

**Keywords:** deformable registration, evaluation, IGRT

## Abstract

Deformable registration has migrated from a research topic to a widely used clinical tool that can improve radiotherapeutic treatment accuracy by tracking anatomical changes. Although various mathematical formulations have been reported in the literature and implemented in commercial software, we lack a straightforward method to verify a given solution in routine clinical use. We propose a metric using concepts derived from vector analysis that complements the standard evaluation tools to identify unrealistic wrappings in a displacement field. At the heart of the proposed procedure is identification of vortexes in the displacement field that do not correspond to underlying anatomical changes. Vortexes are detected and their intensity quantified using the CURL operator and presented as a vortex map overlaid on the original anatomy for rapid identification of problematic regions. We show application of the proposed metric on clinical scenarios of adaptive radiotherapy and treatment response assessment, where the CURL operator quantitatively detected errors in the displacement field and identified problematic regions that were invisible to classical voxel‐based evaluation methods. Unrealistic warping not visible to standard voxel‐based solution assessment can produce erroneous results when the deformable solution is applied on a secondary dataset, such as dose matrix in adaptive therapy or PET data for treatment response assessment. The proposed metric for evaluating deformable registration provides increased usability and accuracy of detecting unrealistic deformable registration solutions when compared to standard intensity‐based approaches. It is computationally efficient and provides a valuable platform for the clinical acceptance of image‐guided radiotherapy.

PACS numbers: 87.57.nj; 87.55.Qr; 87.57.cp

## I. INTRODUCTION

Image‐guided radiation therapy (IGRT) improves treatment accuracy by taking into account anatomical changes occurring during the course of treatment as visualized through repeated imaging. In this approach, a deformable registration algorithm is a key piece of technology that automatically identifies and quantifies changes within the images, with major applications in contouring using atlas segmentation,^1^, ^6^ adaptive radiotherapy,^7^, ^13^ and treatment assessment. Due to its wide applicability, significant improvements in targeting accuracy and, with vendors offering deformable registration algorithms as part of their software solutions, the procedure is expected to be adopted by clinicians as part of standard treatment practice. We contend that for a successful migration of deformable registration algorithms from academic to clinical environments, it is important to establish evaluation guidelines and tools to ensure the procedure can be safely applied without harming the patient.

A deformable registration is in essence an optimization procedure trying to mimic anatomical changes by various mathematical models. In reality, organs in a patient's body will deform under forces exercised between them and from the surrounding medium. Although these forces cannot be measured *in vivo* and are thus unknown, their effect, such as organ compression, inflation, and displacement, can be visualized using different imaging techniques. A deformable registration algorithm estimates the magnitude of these forces by comparing images acquired before and after the deformation. Proposed deformable registration models make various assumptions to solve the problem at hand, being, in essence, synthetic models not necessarily based on underlying anatomy. For example, voxel‐based algorithms^14^, ^15^ assign a vector to each voxel in the input images, and iteratively vary magnitude until the predeformed image — warped with the deformation — matches the postdeformation image. Custom‐made deformable registration models that incorporate anatomical information are possible through the finite element model (FEM) approach.^(16)^ In practice, general settings and assumptions are preferred over highly customized models, but a quality assurance procedure must be in place to ensure that the solution estimated by an algorithm conforms to the expected anatomical forces.

Accuracy assessment depends on the clinical application of the deformable registration algorithm. If the deformation algorithm is used for atlas‐based contouring, voxel motion inside an organ is irrelevant to the algorithm application, with the aim being to correctly match structures borders. A valid approach for this application is to mathematically quantify registration errors by distances between the automated and user‐delineated contours through Haunsdorf or Dice measures.^3^, ^12^, ^13^, ^17^, ^25^ However, for other applications when the resulting displacement field is used to warp supplementary information such as a dose matrix or a PET dataset, it is crucial that the displacement field inside a structure of constant intensity follows real anatomical changes. Voxels in such regions of constant intensity are indiscernible to most deformable registration algorithms,^(25)^ and thus there is little information for the algorithm to build a displacement field that accurately mimics anatomical changes. This is especially important as deformable registration is an ill‐posed inverse problem that does not fulfill Hadamard's postulate on wellposedness, and since a solution may not exist or may not be unique, it is important to verify if the displacement field solution obtained by a deformable registration algorithm correctly models voxel movement.

Indeed, as opposed to rigid registration where only one valid solution is attainable, deformable registration problems might not have a solution in the strict sense, and a solution might not be unique, making their verification problematic. One approach to evaluate a deformable registration technology is to measure accuracy by using markers or clearly visible anatomical locations similar to concepts developed for rigid registration.^8^, ^12^, ^18^, ^26^, ^30^ Such methods are tedious in clinical practice and incomplete when applied to deformable registration because the methods do not validate how the deformation field models voxels movement inside structures of uniform contrast.^(31)^ For either rigid or deformable registration, visual inspection is still the norm, with tools such as the checkerboard or lens used to identify regions of anatomical mismatch. These standard evaluation methods give important visual feedback, but fail to differentiate voxels, and thus do not directly quantify displacement field properties. One academic technique is to warp images with known displacement fields and then inspect the algorithm's ability to recover the deformations.^32^, ^33^ Although this is a rigorous evaluation method, it is a time‐consuming approach, as daily clinical practice lacks the tools and human resources to induce and compare deformations.

We propose, in the following, a metric to complement existing evaluation tools to better understand displacement field properties when correct modeling of anatomical changes is important. At the heart of the proposed procedure is identification of vortexes in the displacement field. Vortexes are a spinning, often turbulent, flow that may develop in fluid mediums. However, patient organs are rather solid and deform smoothly, and as a result, irregular motion descriptors that produce vortexes probably won't truthfully portray the underlying anatomical motion. Vortexes in a displacement field can be identified and characterized using concepts derived from vector analysis and will be used in the following to scan a solution produced by a deformable registration procedure for regions where the displacement is unsmooth. This information is presented to the user as a color‐coded map overlaid on the input images for quick characterization of simulated anatomical behavior.

An overview of the theoretical framework is presented and illustrated with clinical examples where the technique was instrumental in extracting complimentary information from the deformation field. The protocol is easy to implement and use in clinical practice as it does not require phantom measurements, and is applicable to any type of deformable registration algorithm.

## II. MATERIALS AND METHODS

### A. Deformable registration algorithm output

Technically, deformable registration is an optimization problem that searches for a point‐wise transformation minimizing discrepancies between the two datasets to be matched using global voxel‐based similarity metrics. The optimization strategy varies based on the representation of the transformation and the number of variables, being either parametric or nonparametric. In the case of a parametric representation, a finite number of parameters allow representation of nonrigid transformations with realistic complexity. The most common approach in this category is the B‐Spline model where the deformation is defined only on a sparse lattice of nodes overlaid on the image, and the displacement at any voxel is obtained by interpolation from closest lattice nodes. In nonparametric approaches, displacement vectors at any voxel are considered separately to define a dense mapping, with the disadvantage that the optimization space tends to be very large, such that a variational method must replace the optimization.

All deformable registration algorithms use a form of regularization that ensures the resulting displacement field is smooth to correctly describe intra‐ or interfractional anatomical changes. Simply stated, the purpose of the regularization is to ensure that neighboring voxels are mapped to similar location by the displacement field. Without regularization, each voxel is allowed to map freely to any location, a situation that may lead to the case when neighboring voxels are mapped to a completely different location. Such displacement field applied on tissue would create unrealistic folding and tearing. In the case of the B‐Spline approach, this is accomplished by the interpolation between the control nodes. In the case of demons algorithms, an optional smoothing of the current displacement field is performed after each iteration.

**Figure 1:**
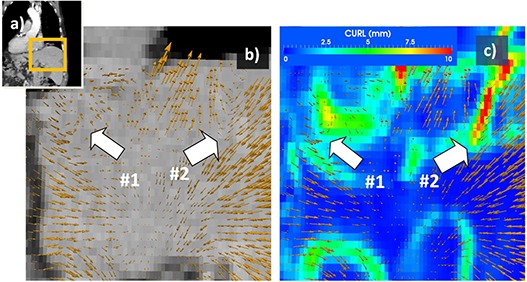
Vortex map used to identify unrealistic motion. Displacement field when mapping exhale and inhale datasets of a 4D CT scan (a) is represented in (b) with yellow arrows, their direction and intensity proportional with displacement and describing motion during the breathing cycle. At region #1, voxels engage in a circular motion, while in region #2 voxels inside the liver suddenly change direction. Neither displacement represents expected anatomical motion and both are identified as unnatural by the vortex map (c).

Independent of a particular implementation of a deformable registration algorithm, its result is, in essence, a vector field that maps voxels between two images. This displacement field can be visualized as a set of vectors positioned at regular grid points as shown in Fig. 1, with arrow direction and length proportional with vector direction and magnitude. Each point in the displacement field has associated a vector $\vec{u}=u\hat{i}+v\hat{j}+w\hat{k}$ with *(u, v, w)* being the displacement values along the *(î, ĵ, ˆ*) normal vectors.

### B. Vortexes

A vector field's rate of rotation in a particular point, sometimes called vorticity, can be mathematically detected by the CURL operator. For a vector field, the operator is associated with the microscopic circulation at a point and is defined as:

(1) $$\nabla \times \vec{u}=\left(\frac{\partial w}{\partial y}‐\frac{\partial v}{\partial z}\right)\hat{i}+\left(\frac{\partial u}{\partial z}‐\frac{\partial w}{\partial x}\right)\hat{j}+\left(\frac{\partial v}{\partial x}‐\frac{\partial u}{\partial y}\right)\hat{k}$$

Technically speaking, the operator measures changes in directions between two neighboring vectors in the displacement field. If the displacement field is smooth and consistent with expected anatomical motion inside an organ, changes between neighboring vectors are minimal and CURL values are low. If the displacement field is fragmented with neighboring vectors mapping in different directions, values of the CURL operator are high.

This concept as applied in radiotherapy is illustrated visually in Fig. 1, where the operator identifies discontinuities in the displacement field on a registration between inhale and exhale of 4D datasets. The displacement field in the liver regions is highlighted in (Fig. 1b) as a vector map. The regions marked with white arrows mark displacement field inconsistencies. At arrow #1, vectors engage in a circular motion. In reality, applying such a field on the organ would produce a vortex of its voxels. At arrow #2, for example, some vectors are directed toward the liver center, while neighboring arrows point outward. In reality, applying such a field on a solid organ such as the liver would produce organ disruption.

Vortexes in the displacement field detected by the CURL operator and characterizing such unnatural behavior are shown in (Fig. 1c) as a color‐coded map placed in the same coordinate system with the vector field visualization. Vortex intensity is color‐coded from blue, corresponding to small intensities, to the highest intensities shown in red. Inaccuracies observed in the visual inspection of this particular solution are automatically marked by the CURL operator.

## III. RESULTS

### A. Phantom case

**Figure 2:**
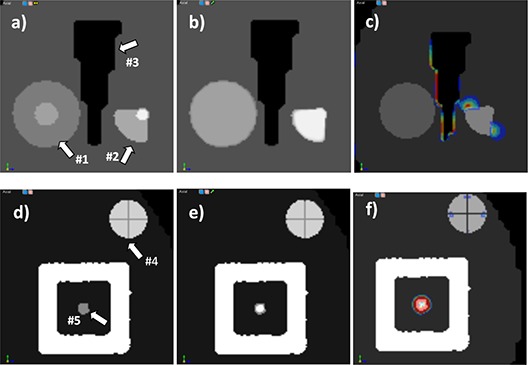
Standard versus displacement field‐based measures for the phantom experiment for phantom inserts mimicking tumor shrinkage (upper row) or artifacts (lower row). Superimposed images of the input datasets are shown in (a) and (d), while results assessed with standard evaluation tools are shown in (b) and (e). Complementing the standard image‐based assessment, measures derived from the displacement field provide additional information on the nature of the resulting solution. Discontinuities in the displacement field are identified by the CURL operator in (c) and (f). See for a detailed comparison of field and measure features.

To exemplify and verify the proposed QA metrics, we constructed a synthetic phantom with known characteristics modeled after a Quasar Body phantom (Modus Medical Devices, London, ON),^(34)^ a Lucite phantom designed for nondosimetric quality assurance. CT images of the phantom were obtained using a GE LightSpeed scanner (GE Medical Systems, Milwaukee, WI) with a 2.5 mm slice thickness. Various phantom inserts consisting of standard cylinders and cubes were segmented, and synthetic CT datasets were created by warping these segmentations with known deformations and defects, followed by conversion of segmentations to voxels of constant HU units. As depicted in (Figs. 2a)and (2d), structures marked with 1 and 2 were expanded to mimic tumor shrinkage. The figure shows the fixed dataset superimposed on the moving dataset, where synthetic differences are marked with arrows and can be observed as gray regions. (Figure 2d) illustrates in a similar display mimicking artifacts occurring in typical clinical CBCT datasets. The defects are a crosshair‐like streak artifact marked with arrow #4 and an inaccurate HU artifact marked with arrow #5. Registration of the original and modified datasets was performed using a diffeomorphic demons‐type^(35)^ algorithm and the displacement field was analyzed using the proposed metrics.

**Figure 3:**
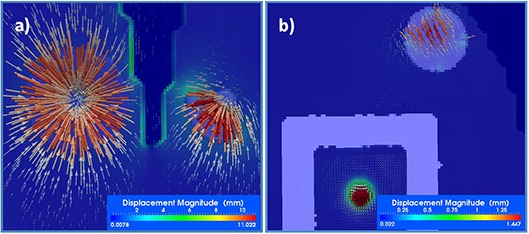
Details of different inserts in the phantom experiment, comparing the displacement field, shown as scaled vectors, with the vortex map, shown as a color‐coded overlay in the background. The vortex map correctly identifies sudden changes in the displacement field.

As shown in (Figs. 2c)–2(f), the CURL operator gives a better understanding of deformation field characteristics that complement the simple image‐based assessment by quantifying the behavior of the displacement field. In the standard evaluation method showing target and result ((Figs. 2b)and (2d), 2(c) and 2(f)) images are almost identical, with few visible differences, as the algorithm was able to mimic structure shrinkage. Contractions are observed in structures 1 and 2, as the deformation field tries to inflate the voxels in the small initial volume to match its larger size in the target image. At the same time, the field must preserve shape number 3, creating a compression as the deformation is stopped from expansion in this structure's vicinity. The vortex map ranges from 0 to 5.1 and is shown in (Fig. 2e), with strong hot spots in structures 4 and 5, where the induced artifacts are present. For a better understanding of these measures, the displacement field on these structures is shown as arrows in Fig. 3, with arrow direction and color according to displacement field intensity and direction. On structure 1 ((Fig. 3a), arrows point to the center, indicating a constant field compression. For comparison, compression on structure 2 is nonuniform. For structure 3 ((Fig. 3b) large changes in the displacement field are observed due to the small round artifacts in its middle that must be created by the deformation field from the neighboring voxels. These discrepancies are detected and marked by the CURL operator. As is obvious in this figure, visually inspecting the deformation field using arrows to mimic displacements would be tedious, because too many vectors are present, but the proposed operators automatically detect characteristics and provide a useful tool to quantify the displacement field behavior.

### B. Deformable registration for adaptive radiotherapy

Integration of CBCT imaging data and dose tracking for radiation therapy has drawn the interest of many clinicians because of its compelling advantages in defining tumor volume and designing treatment plans that better spare critical structures. In this approach, information obtained from online cone‐beam CT (CBCT) scans probe patient anatomy that can be used to assess the dose delivered to date and make plan adaptations, if needed. Deformable image registration is used to map daily dose distributions to a common coordinate system, typically the original treatment plan, to create a cumulative dose distribution. The approach can be used to estimate the difference between the planned and delivered doses^(36)^ or to study the need for re‐planning.^37^, ^38^

We mapped the dose to the CBCT datasets using two algorithms, a diffeomorphic demons algorithm^(36)^ and a B‐Spline^(15)^ algorithm, both implemented in the ITK library,^(39)^ for a case of a large tumor in the pelvis expected to change in size and shape during treatment, treated to an initial dose of 45 Gy. The diffeomorphic demons algorithm is a monomodality algorithm that implicitly assumes a structure is represented by voxels of the same intensity in both datasets.

This is not necessarily valid, as various artifacts and an increased level of noise are present in the CBCT dataset due to acquisition geometry. As compared with the standard multidetector CT, images acquired on a cone‐beam CT (CBCT) dataset have increased scatter incoming on the detector that implicitly produces artifacts, decreased contrast‐to‐noise, and inaccuracies in CT number calculations in the reconstructed images. In addition, high density materials in the field of view that highly attenuate the X‐ray beam making the attenuation, values of objects behind the object are incorrectly high causing streak artifacts.

**Figure 4:**
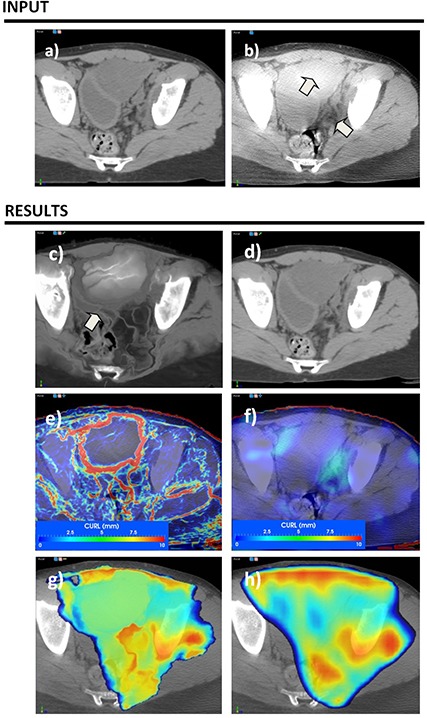
Evaluation of deformable registration as applied in adaptive radiotherapy. The CT dataset (a) was warped to the CBCT dataset (b) using a monomodality diffeomorphic demons algorithm (c), (left column in results section) and a multimodality B‐Spline algorithm (d), (right column in results section). In (c), HU calibrations and various artifacts present in the CBCT dataset violated the monomodality assumption of the first algorithm, leading to unrealistic warping. In (d), the solution provided by algorithm 2 interpolates artifacts but is not able to model small local organ changes. The vortex map easily catches these characteristics of the displacement fields (e) and (f). When warping the dose distribution from the planning to the CBCT dataset, the distribution warped by algorithm 1 looks unnatural and unrealistic (g), while the dose distribution warped by algorithm 2 looks natural (h).

**Figure 5:**
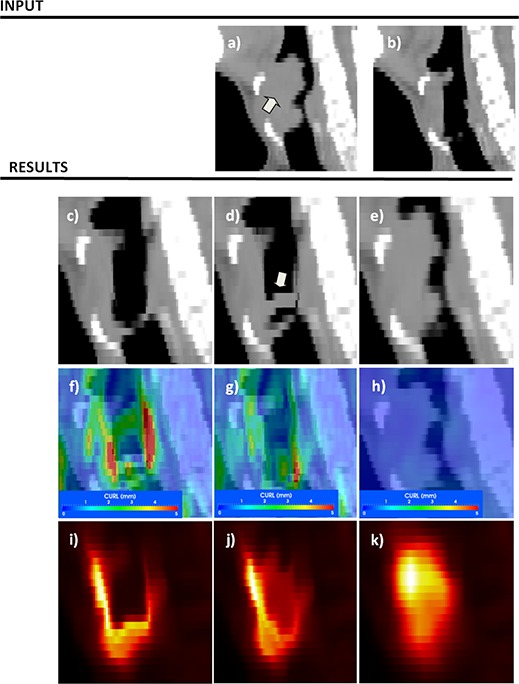
Evaluation of deformable registration as applied to response assessment. The CT component of pre‐ and post‐treatment PET scans is used for the registration and the resulting transform is applied on the SUV values for response assessment. Although algorithm 1, depicted in the left column, provides the best match voxel‐wise, clinically, algorithm 3 is more suitable to describe anatomical changes. Pre‐ and post datasets are shown in (a) and (b), while each column in the results section illustrates a different deformable registration algorithm.

A simple visual comparison of the original CT and CBCT datasets presented in Fig. 4, upper row, illustrates a visible hot spot in the bladder and a darker region in the soft muscle. For the demons diffeomorphic algorithm, these artifacts, combined with the low parameter of the regularization term in the algorithm's mathematics, produced the result shown in (Fig. 4c), which is correct from the viewpoint of metrics because previously mentioned hot and cold spots are mimicked in the resulting transform by warping voxels from bone or air. However, this is an unrealistic solution from a clinical point of view. For comparison, a multimodality B‐Spline algorithm with a few control nodes to provide strong regularization is shown in (Fig. 4d). In this solution, although the displacement field is uniform, small changes in bladder shape (marked with arrow) are not necessarily modeled. The vortex map, presented in the third row of Fig. 4 for both algorithms, identifies many vortexes with high intensity in the demons solution, ranging from 0 to 67.34, while the B‐Spline solution has only a few small vortexes with values ranging from 0 to 6.86 in soft tissue and away from the target. When applying these solutions on the dose matrix, the dose mapped through the B‐Spline ((Fig. 4e) appears smooth, while the dose mapped with the demons algorithm appears jagged and unrealistic ((Fig. 4d). The bad solution presented in (Fig. 4c) is an extreme case, easily identifiable by visual inspection; however, most solutions we encountered in clinical practice have some unrealistic warping on local voxels that are not easily identifiable by visual inspection. For most clinical solutions, the vortex map was helpful in automatically identifying regions of nonsmooth displacements. Figure 5 illustrates such a case when warping of similar magnitude is not identifiable by visual inspection only.

Similar to IMRT QA, where the gamma index is used to measure discrepancies (medical physicist decides whether high values may influence treatment based on their locations and intensities), in the proposed evaluation method, the physician decides if vortexes are clinically relevant or justified. For example in Fig. 1, vortexes describing irregular motion are justifiable on the lung–diaphragm interface, as these organs move independently, but are not clinically justifiable inside the liver. Vortexes far away from regions of clinical interest, such as tumor or critical organs, may be acceptable, as long as the dose or other measures are not computed in the affected area.

### C. Deformable registration for treatment assessment

In Figure 5, we show usage of the vortex map to identify errors when deformable registration is used in treatment response assessment to compare pre‐ and post‐treatment positron emission (PET) datasets.^40^, ^41^ In this approach, the baseline PET/CT scan is acquired up to a week before the start of the chemotherapeutic or radiotherapeutic treatment. The follow‐up PET/CT scan is acquired in the first half of therapy, typically after 1 to 2 weeks of chemotherapy. Treatment efficiency is then assessed by comparing changes between the two datasets.^42^, ^43^ As changes in SUV intensities are used to monitor treatment process, it is essential to discern changes induced by the treatment itself from any other nontreatment‐related factors. Deformable registration is needed to account for organ displacements and posture changes.

Application of the QA procedure when deformable registration was used for treatment assessment is presented in Fig. 5. Deformable registration input included CT attenuation components of the PET scans containing clearly visible anatomical information, but registration result was further applied on the PET component for SUV comparison. Input datasets are in (Figs. 5a) and (b), with an arrow marking the original tumor location, visible in the pretreatment dataset, but significantly reduced on the aftertreatment scan. Three solutions spanning the trade‐off between smoothness of the deformation field and ability to model small changes are compared for this task: a diffeomorphic demons^(44)^ algorithm with the smoothing displacement field option turned off (left column), the same algorithm with the same option turned on (middle panel), and a B‐Spline algorithm^(15)^ (right panel) using just seven grid nodes per dimension.

Solutions obtained by the three algorithms are presented in (Figs. 5c), (d), and (e). In a standard visual evaluation comparing the result with the target (post‐treatment scan), the first algorithm is highly successful in modeling changes as the two compared images are almost alike ((Fig. 5c). The smooth version of the same algorithm obtains a good result, with a small discrepancy marked with an arrow ((Fig. 5d), while the B‐Spline algorithm provides the worst result as it is not able to match tumor changes ((Fig. 5e).

When turning the vortex map on, the first two solutions present high‐intensity vortexes near the tumor location (26.92 and 26.64 for each solution), while last solution presents just a few vortexes ranging from 0 to 0.65 ((Fig. 5f), (g), (h)). The SUV values warped with these three solutions are presented in the lower row of Fig. 5. Although solutions 1 and 2 are very similar in terms of warping the CT scan, when applied to the PET component, the SUV intensity is visibly different ((Figs. 5i), (j)). Clinically, the solution provided by the third algorithm is the worst in terms of registration metric, but in a visual inspection of the vortex field it appears as the most accurate for treatment response assessment. The first two solutions warp the tumor to the healthy esophagus wall. However, cells in the tumor are destroyed by radiation and eliminated from the patient's body. Thus the high‐intensity SUV values should not be mapped to the healthy esophagus, but rather kept at their original coordinates.

## IV. DISCUSSION

We show that for adaptive radiotherapy mapping additional datasets such as dose or PET datasets, careful case‐by‐case inspection of algorithm accuracy in describing anatomical changes is essential to assure safe and accurate practice of radiotherapy, by identifying in a specific solution, regions where the deformation field is unrealistic. Deformation field can be scanned to find vortexes in the displacement field. These regions should match clinical expectations. Measures proposed in this work can be used in conjunction with standard visualization tools to qualitatively characterize the deformation field for a specific solution obtained on real patient images. While not sufficient by themselves in detecting deformable registration errors, the tool presented in this report helps identify suspicious locations where the displacement field may not properly describe the expected anatomical change.

In clinical practice, there isn't any circular soft‐tissue rotation expected and therefore the CURL values should be ideally zero. In our examples, we noticed that clinically valid solutions have CURL values ranging from 0 to 5 mm, while nonphysical solutions had CURL values from 5 to 10, with lower values recorded in regions of smooth transitions and voided of artifacts, and values above 3 observed in regions where the algorithm struggled to match the image datasets. However, a larger study on datasets from different anatomical sites is needed to validate clinically these initial findings. In the cases presented, the operator was useful in detecting regions where the displacement field had high values as compared with other regions in the solution. These regions of high CURL values should be far away from clinically important structures, with both their number and intensity being associated with nonphysical solutions.

Previous reports show that registration errors are prone to emerge in regions with uniform image intensity and low‐intensity gradient regions and are not visible with standard voxel‐based methods.^(45)^ Although it is hard to identify differences by inspecting the images directly, analysis of the deformation field through the CURL operator provides complementary information that better identifies and characterizes tissue warping. As shown in Figs. 4 and 5, a vortex map leads to a better understanding of solution properties when compared to standard voxel‐based methods because the displacement field is directly evaluated.

**Figure 6:**
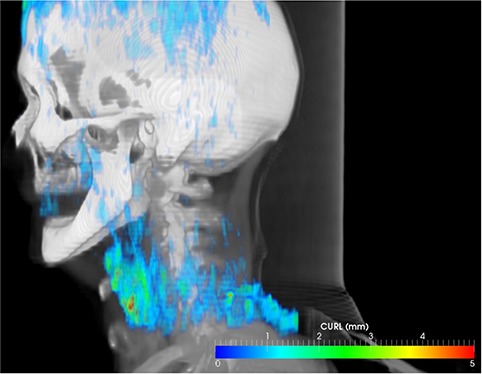
Hot spots in the displacement field can be easily identified using the vortex measure. In this volumetric 3D view, the vortex map is displayed color‐coded in relation to patient anatomy for fast localization of doubtful regions in the displacement field.

Phantom measurements that mimic patient anatomy can be used to assess deformable registration accuracy in specific conditions,^31^, ^45^, ^48^ but are time‐consuming and not well‐suited for routine evaluation, as algorithm settings are usually tailored to image quality,^49^, ^50^ levels of noise,^(51)^ or partial volume effects, with estimations obtained on phantom studies overestimating accuracy if images of lesser quality are used as input. Furthermore, accuracy depends on parameter selection, which is closely associated with intensity gradients of the underlying image,^(45)^ or are site‐specific. For example, the number of iterations or the number of nodes in the B‐Spline model have been found to be site‐specific,^(45)^ with a different number of nodes needed to accurately register the rectum or lung. For a casual user, the evaluation metric presented here provides a tool that quickly analyzes and quantifies a solution obtained under specific settings, and visually guides the user in selecting parameters that work best with the input datasets, as the vortexes map calculation takes less than one second and is easily displayed as a 3D volume or a color‐wash superimposed on the CT dataset (as illustrated in Fig. 6). As a software procedure that extracts relevant clinical information from the displacement field, it is ideally suited for a daily clinical operations where the plausibility of a solution should be verified, reducing the time spent by physics staff on verifications.

This study does not endorse a particular deformable registration algorithm, but rather highlights the need for evaluating, solution‐by‐solution, the results of such an algorithm to gauge if the displacement map provided by a deformable algorithm is plausible from a clinical perspective. In a comprehensive publication, Kashani et al.^(31)^ reported interalgorithm comparison at different institutions and noted that “no algorithm was uniformly accurate across all regions in a phantom”, with larger errors seen in regions of significant shape changes and in areas of uniform contrast. As noted by Zhong et al.,^(45)^ performance in regions with low‐intensity gradients is difficult to assess. This study highlights the need for a direct measure derived from the displacement field when assessing accuracy of deformable warping for clinical applications.

Similar to established IMRT QA procedures that verify leaf position accuracy with devices or back‐up software that is independent of the treatment planning optimization algorithm, the proposed deformable registration QA approach constitutes an independent check that quantifies errors by measures obtained on a specific result, independent of its generating algorithm. The proposed measure is also useful in discarding solutions that are mathematically valid by the metric function, but produce clinically unrealistic warping.

## V. CONCLUSIONS

There is a lack of efficient evaluation tools to aid the casual user to identify unrealistic warping within a deformable registration solution. Such a tool should be simple to use in clinical practice, preferably without the use of markers and phantoms, and should be algorithm‐independent. Our proposed metric fulfills these requirements. The procedure relies on extracting displacement field characteristics that are overlaid on the original anatomy for quick identification of problematic regions. Analysis of a displacement field through the CURL operator is ideally suited to evaluate the deformable registration when accuracy of its resulting displacement field inside a structure is vital, as the operator identifies nonrealistic displacement that is invisible in standard voxel‐based approaches, providing an important tool to successful implementation of adaptive radiotherapy in clinical practice.

## References

[1] Reed VK , Woodward WA , Zhang L , et al. Automatic segmentation of whole breast using atlas approach and deformable image registration. Int J Radiat Oncol Biol Phys. 2009;73(5):1493–500. 18804333 10.1016/j.ijrobp.2008.07.001 PMC2729433

[2] Wang H , Garden AS , Zhang L , et al. Performance evaluation of automatic anatomy segmentation algorithm on repeat or four‐dimensional computed tomography images using deformable image registration method. Int J Radiat Oncol Biol Phys. 2008;72(1):210–19. 18722272 10.1016/j.ijrobp.2008.05.008 PMC2593788

[3] Wijesooriya K , Weiss E , Dill V , et al. Quantifying the accuracy of automated structure segmentation in 4D CT images using a deformable image registration algorithm. Med Phys. 2008;35(4):1251–60. 18491517 10.1118/1.2839120 PMC2811553

[4] Thornqvist S , Petersen JB , Hoyer M , Bentzen LN , Muren LP . Propagation of target and organ at risk contours in radiotherapy of prostate cancer using deformable image registration. Acta Oncol. 2010;49(7):1023–32. 20831491 10.3109/0284186X.2010.503662

[5] Ellingsen LM , Chintalapani G , Taylor RH , Prince JL . Robust deformable image registration using prior shape information for atlas to patient registration. Comput Med Imaging Graph. 2010;34(1):79–90. 19515532 10.1016/j.compmedimag.2009.05.003 PMC2990688

[6] Schreibmann E , Chen GT , Xing L . Image interpolation in 4D CT using a BSpline deformable registration model. Int J Radiat Oncol Biol Phys. 2006;64(5):1537–50. 16503382 10.1016/j.ijrobp.2005.11.018

[7] Lee C , Langen KM , Lu W , et al. Assessment of parotid gland dose changes during head and neck cancer radiotherapy using daily megavoltage computed tomography and deformable image registration. Int J Radiat Oncol Biol Phys. 2008;71(5):1563–71. 18538505 10.1016/j.ijrobp.2008.04.013

[8] Nithiananthan S , Brock KK , Daly MJ , Chan H , Irish JC , Siewerdsen JH . Demons deformable registration for CBCT‐guided procedures in the head and neck: convergence and accuracy. Med Phys. 2009;36(10):4755–64. 19928106 10.1118/1.3223631 PMC2771717

[9] Zhang G , Huang TC , Feygelman V , Stevens C , Forster K . Generation of composite dose and biological effective dose (BED) over multiple treatment modalities and multistage planning using deformable image registration. Med Dosim. 2010;35(2):143–50. 19931027 10.1016/j.meddos.2009.05.001

[10] Velec M , Moseley JL , Eccles CL , et al. Effect of breathing motion on radiotherapy dose accumulation in the abdomen using deformable registration. Int J Radiat Oncol Biol Phys. 2011;80(1):265–72. 20732755 10.1016/j.ijrobp.2010.05.023 PMC3010501

[11] Hasan Y , Kim L , Wloch J , et al. Comparison of planned versus actual dose delivered for external beam accelerated partial breast irradiation using cone‐beam ct and deformable registration. Int J Radiat Oncol Biol Phys. 2011;80(5):1473–76. 20656415 10.1016/j.ijrobp.2010.04.013

[12] Ostergaard Noe K , De Senneville BD , Elstrom UV , Tanderup K , Sorensen TS . Acceleration and validation of optical flow based deformable registration for image‐guided radiotherapy. Acta Oncol. 2008;47(7):1286–93. 18661435 10.1080/02841860802258760

[13] Castadot P , Lee JA , Parraga A , Geets X , Macq B , Gregoire V . Comparison of 12 deformable registration strategies in adaptive radiation therapy for the treatment of head and neck tumors. Radiother Oncol. 2008;89(1):1–12. 18501456 10.1016/j.radonc.2008.04.010

[14] Wang H , Dong L , O'Daniel J , et al. Validation of an accelerated ‘demons’ algorithm for deformable image registration in radiation therapy. Phys Med Biol. 2005;50(12):2887–905. 15930609 10.1088/0031-9155/50/12/011

[15] Mattes D , Haynor DR , Vesselle H , Lewellen TK , Eubank W . PET‐CT image registration in the chest using free‐form deformations. IEEE Trans Med Imaging. 2003;22(1):120–28. 12703765 10.1109/TMI.2003.809072

[16] Brock KK , Sharpe MB , Dawson LA , Kim SM , Jaffray DA . Accuracy of finite element model‐based multi‐organ deformable image registration. Med Phys. 2005;32(6):1647–59. 10.1118/1.1915012 16013724

[17] Makni N , Puech P , Lopes R , Dewalle AS , Colot O , Betrouni N . Combining a deformable model and a probabilistic framework for an automatic 3D segmentation of prostate on MRI. Int J Comput Assist Radiol Surg. 2009;4(2):181–88. 20033618 10.1007/s11548-008-0281-y

[18] Suh JW , Wyatt CL . Deformable registration of supine and prone colons for computed tomographic colonography. J Comput Assist Tomogr. 2009;33(6):902–11. 19940658 10.1097/RCT.0b013e3181a7e2c1

[19] Bender ET , Mehta MP , Tome WA . On the estimation of the location of the hippocampus in the context of hippocampal avoidance whole brain radiotherapy treatment planning. Technol Cancer Res Treat. 2009;8(6):425–32. 19925026 10.1177/153303460900800604 PMC2797122

[20] Hwang AB , Bacharach SL , Yom SS , et al. Can positron emission tomography (PET) or PET/Computed Tomography (CT) acquired in a nontreatment position be accurately registered to a head‐and‐neck radiotherapy planning CT? Int J Radiat Oncol Biol Phys. 2009;73(2):578–84. 19084350 10.1016/j.ijrobp.2008.09.041

[21] Chao M , Xie Y , Xing L . Auto‐propagation of contours for adaptive prostate radiation therapy. Phys Med Biol. 2008;53(17):4533–42. 18677041 10.1088/0031-9155/53/17/005 PMC12121639

[22] Chao M , Li T , Schreibmann E , Koong A , Xing L . Automated contour mapping with a regional deformable model. Int J Radiat Oncol Biol Phys. 2008;70(2):599–608. 18207035 10.1016/j.ijrobp.2007.09.057

[23] Orban de Xivry J , Janssens G , Bosmans G , et al. Tumour delineation and cumulative dose computation in radiotherapy based on deformable registration of respiratory correlated CT images of lung cancer patients. Radiother Oncol. 2007;85(2):232–38. 17936388 10.1016/j.radonc.2007.08.012

[24] Bender ET and Tomé WA . The utilization of consistency metrics for error analysis in deformable image registration. Phys Med Biol. 2009;54(18):5561–77. 19717890 10.1088/0031-9155/54/18/014 PMC2798737

[25] Lawson JD , Schreibmann E , Jani AB , Fox T . Quantitative evaluation of a cone‐beam computed tomography‐planning computed tomography deformable image registration method for adaptive radiation therapy. J Appl Clin Med Phys. 2007;8(4):2432. 18449149 10.1120/jacmp.v8i4.2432 PMC5722621

[26] Brock KK . Results of a multi‐institution deformable registration accuracy study (MIDRAS). Int J Radiat Oncol Biol Phys. 2010;76(2):583–96. 19910137 10.1016/j.ijrobp.2009.06.031

[27] Brock KK , Nichol AM , Menard C , et al. Accuracy and sensitivity of finite element model‐based deformable registration of the prostate. Med Phys. 2008;35(9):4019–25. 18841853 10.1118/1.2965263

[28] Miyabe Y , Narita Y , Mizowaki T , et al. New algorithm to simulate organ movement and deformation for four‐dimensional dose calculation based on a three‐dimensional CT and fluoroscopy of the thorax. Med Phys. 2009;36(10):4328–39. 19928063 10.1118/1.3213083

[29] Nithiananthan S , Brock KK , Irish JC , Siewerdsen JH . Deformable registration for intraoperative cone‐beam CT guidance of head and neck surgery. Conf Proc IEEE Eng Med Biol Soc. 2008;2008:3634–37. 19163498 10.1109/IEMBS.2008.4649995

[30] Wu Z , Rietzel E , Boldea V , Sarrut D , Sharp GC . Evaluation of deformable registration of patient lung 4DCT with subanatomical region segmentations. Med Phys. 2008;35(2):775–81. 18383700 10.1118/1.2828378

[31] Kashani R , Hub M , Balter JM , et al. Objective assessment of deformable image registration in radiotherapy: a multi‐institution study. Med Phys. 2008;35(12):5944–53. 19175149 10.1118/1.3013563 PMC2673610

[32] Schreibmann E , Xing L . Narrow band deformable registration of prostate magnetic resonance imaging, magnetic resonance spectroscopic imaging, and computed tomography studies. Int J Radiat Oncol Biol Phys. 2005;62(2):595–605. 15890605 10.1016/j.ijrobp.2005.02.001

[33] Lu W , Chen ML , Olivera GH , Ruchala KJ , Mackie TR . Fast free‐form deformable registration via calculus of variations. Phys Med Biol. 2004;49(14):3067–87. 15357182 10.1088/0031-9155/49/14/003

[34] Craig T , Brochu D , Van Dyk J . A quality assurance phantom for three‐dimensional radiation treatment planning. Int J Radiat Oncol Biol Phys. 1999;44(4):955–66. 10386655 10.1016/s0360-3016(99)00070-x

[35] Vercauteren T , Pennec X , Perchant A , Ayache N . Non‐parametric diffeomorphic image registration with the demons algorithm. Med Image Comput Comput Assist Interv. 2007;10(Pt 2):319–26. 10.1007/978-3-540-75759-7_39 18044584

[36] O'Daniel JC , Garden AS , Schwartz DL , et al. Parotid gland dose in intensity‐modulated radiotherapy for head and neck cancer: is what you plan what you get? Int J Radiat Oncol Biol Phys. 2007;69(4):1290–96. 17967319 10.1016/j.ijrobp.2007.07.2345 PMC2288571

[37] Olteanu LA , Madani I , De Neve W , Vercauteren T , De Gersem WI . Evaluation of deformable image coregistration in adaptive dose painting by numbers for head and neck cancer. Int J Radiat Oncol Biol Phys. 2012;83(2):696–703. 22153223 10.1016/j.ijrobp.2011.07.037

[38] Duprez F , De Neve W , De Gersem W , Coghe M , Madani I . Adaptive dose painting by numbers for head‐and‐neck cancer. Int J Radiat Oncol Biol Phys. 2011;80(4):1045–55. 20643512 10.1016/j.ijrobp.2010.03.028

[39] Yoo TS . Insight into images: principles and practice for segmentation, registration, and image analysis: Wellesey, MA: A K Peters; 2004.

[40] Robbins RJ , Wan Q , Grewal RK , et al. Real‐time prognosis for metastatic thyroid carcinoma based on 2‐[18F]fluoro‐2‐deoxy‐D‐glucose‐positron emission tomography scanning. J Clin Endocrinol Metab. 2006;91(2):498–505. 16303836 10.1210/jc.2005-1534

[41] Weber WA . Positron emission tomography as an imaging biomarker. J Clin Oncol. 2006;24(20):3282–92. 16829652 10.1200/JCO.2006.06.6068

[42] Juweid ME , Stroobants S , Hoekstra OS , et al. Use of positron emission tomography for response assessment of lymphoma: Consensus of the Imaging Subcommittee of International Harmonization Project in Lymphoma. J Clin Oncol. 2007;25(5):571–78. 17242397 10.1200/JCO.2006.08.2305

[43] Weber WA , Czernin J , Phelps ME , Herschman HR . Technology insight: novel imaging of molecular targets is an emerging area crucial to the development of targeted drugs. Nat Clin Pract Oncol. 2008;5(1):44–54. 18097456 10.1038/ncponc0982 PMC2830564

[44] Vercauteren T , Pennec X , Perchant A , Ayache N . Diffeomorphic demons: efficient non‐parametric image registration. Neuroimage. 2009;45(1 Suppl):S61–S72. 19041946 10.1016/j.neuroimage.2008.10.040

[45] Zhong H , Kim J , Chetty IJ . Analysis of deformable image registration accuracy using computational modeling. Med Phys. 2010;37(3):970–79. 20384233 10.1118/1.3302141 PMC3188658

[46] Chang J , Suh TS , Lee DS . Development of a deformable lung phantom for the evaluation of deformable registration. J Appl Clin Med Phys. 2010;11(1):3081. 20160694 10.1120/jacmp.v11i1.3081 PMC5719763

[47] Chao M , Schreibmann E , Li T , Wink N , Xing L . Automated contour mapping using sparse volume sampling for 4D radiation therapy. Med Phys. 2007;34(10):4023–29. 17985648 10.1118/1.2780105

[48] Kashani R , Hub M , Kessler ML , Balter JM . Technical note: a physical phantom for assessment of accuracy of deformable alignment algorithms. Med Phys. 2007;34(7):2785–88. 17821985 10.1118/1.2739812

[49] Boldea V , Sharp GC , Jiang SB , Sarrut D . 4D‐CT lung motion estimation with deformable registration: quantification of motion nonlinearity and hysteresis. Med Phys. 2008;35(3):1008–18. 18404936 10.1118/1.2839103

[50] Martel AL , Froh MS , Brock KK , Plewes DB , Barber DC . Evaluating an optical‐flow‐based registration algorithm for contrast‐enhanced magnetic resonance imaging of the breast. Phys Med Biol. 2007;52(13):3803–16. 17664578 10.1088/0031-9155/52/13/010

[51] Paquin D , Levy D , Xing L . Multiscale deformable registration of noisy medical images. Math Biosci Eng. 2008;5(1):125–44. 18193935 10.3934/mbe.2008.5.125

